# Simultaneous Magnetic Resonance Imaging and Consolidation Measurement of Articular Cartilage

**DOI:** 10.3390/s140507940

**Published:** 2014-05-05

**Authors:** Robert Mark Wellard, Jean-Philippe Ravasio, Samuel Guesne, Christopher Bell, Adekunle Oloyede, Greg Tevelen, James M. Pope, Konstantin I. Momot

**Affiliations:** 1 Discipline of Chemistry, Physics and Mechanical Engineering, Queensland University of Technology/2 George St., Brisbane 4001, Australia; 2 SonoSite France Sarl, 19, avenue de Norvege, Parc d'activite les Fjords, Immeuble le Vega, 91140 Villebon sur Yvette, France; E-Mail: jp.ravasio@free.fr; 3 National Instruments France, 2 rue Hennape, 92735 Nanterre Cédex, France; E-Mail: samuelguesne@hotmail.fr; 4 Discipline of Biomedical Engineering and Medical Physics, Queensland University of Technology/2 George St., Brisbane 4001, Australia; E-Mails: Christopher.Bell@csiro.au (C.B.); k.oloyede@qut.edu.au (A.O.); j.pope@qut.edu.au (J.M.P.); k.momot@qut.edu.au (K.I.M.); 5 Science and Engineering Faculty, Queensland University of Technology/2 George St., Brisbane 4001, Australia; E-Mail: gregtev@gmail.com

**Keywords:** fiber interferometers, magnetic resonance imaging, compression, collagen structure, consolidometery

## Abstract

Magnetic resonance imaging (MRI) offers the opportunity to study biological tissues and processes in a non-disruptive manner. The technique shows promise for the study of the load-bearing performance (consolidation) of articular cartilage and changes in articular cartilage accompanying osteoarthritis. Consolidation of articular cartilage involves the recording of two transient characteristics: the change over time of strain and the hydrostatic excess pore pressure (HEPP). MRI study of cartilage consolidation under mechanical load is limited by difficulties in measuring the HEPP in the presence of the strong magnetic fields associated with the MRI technique. Here we describe the use of MRI to image and characterize bovine articular cartilage deforming under load in an MRI compatible consolidometer while monitoring pressure with a Fabry-Perot interferometer-based fiber-optic pressure transducer.

## 1. Introduction

**Figure 1.**
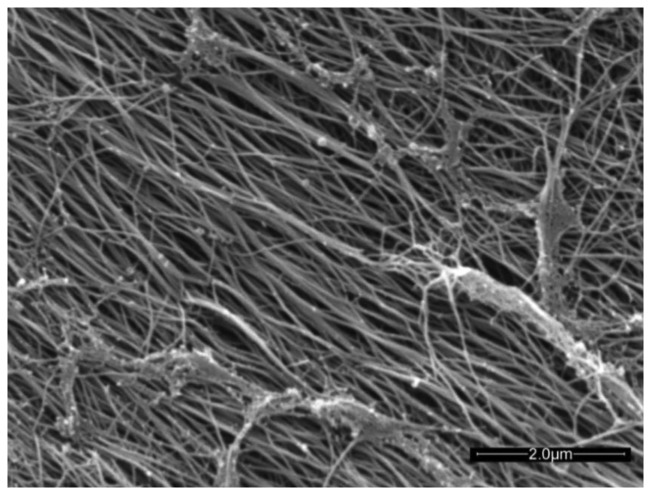
Scanning electron micrograph showing the high degree of collagen fiber alignment in articular cartilage, 30,000× magnification (reprinted with permission from [2]).

Articular cartilage (AC) is an avascular connective tissue lining the articulating surfaces of long bones in mammals. Healthy adult human cartilage is 2–4 mm thick and comprises extracellular biopolymers (collagen, 15%–20%; proteoglycans, 3%–10%; and lipids, 1%–5%) with the primary function of enabling load bearing in a mobile joint. The structural collagen component of cartilage is highly aligned, being normal to the supporting bone surface and arching to a parallel alignment at the synovial surface [1] (see Figure 3 in [1]). The highly aligned nature of the collagen fibers can be seen in the scanning electron micrograph of trypsin treated cartilage in Figure 1.

AC is both structurally heterogenous and mechanically anisotropic [3]. The anisotropy is associated with collagen fiber alignment, which varies throughout the thickness of the cartilage [1,4] and the distribution of hydrated proteoglycans [5], which together generate the osmotic pressure-based load bearing stiffness of the tissue. Under static compression, AC exhibits a complex behavior referred to as consolidation [6], with an initial rapid increase in strain to a plateau, which is maintained. At the same time a proportion of the load is transferred to the tissue fluid, resulting in a rise in hydrostatic excess pore pressure (HEPP) to a maximum, after which the fluid pressure decays as the solid component of the AC bears more of the applied load. The equilibrium between compressive tissue deformation and out-flow of extracellular water is maintained by an increase in osmotic pressure that resists the outflow of tissue water [6,7]. In functionally compromised states such as osteoarthritis, the degradation and loss of proteoglycans in AC results in a loss of mechanical function and eventual degradation of the tissue as a whole. This, in turn, leads to reduced patient mobility and is responsible for increased health-care costs to the individual and the community. With a prevalence of 13.9% in adults aged 25 years and older [8], costs of osteoarthritis were estimated to be $185.5 billion using 1996–2005 data from the USA [9] and account for 1%–2.5% of gross national product in the USA, Canada, UK, France, and Australia [10]. Understanding of the characteristics responsible for the load bearing efficiency of AC and the factors leading to its degradation is incomplete. The importance of water and its local environment in load bearing by cartilage makes it an ideal candidate for study by magnetic resonance imaging (MRI), a technique that is sensitive to the mobility of water molecules in biological tissue. MRI is a technique that is ideally suited to the study of structural changes in soft tissues such as AC [11–13], with a range of characterization techniques available [3,4,11,12,14].

MRI utilizes the magnetic moments of certain nuclei, such as ^1^H, which partially align with a strong magnetic field, resulting in a net nuclear magnetization of the sample. Brief irradiation with an appropriate radiofrequency pulse tips the magnetization vector out of its equilibrium direction; this is known as RF excitation. Excited magnetization undergoes two dynamic processes: (1) its transverse component precesses, inducing a current in a tuned radiofrequency coil; and (2) the magnetization vector gradually returns to equilibrium. The decay of the MRI signal as the sample returns to equilibrium is associated with characteristic time constants, *T*_1_ and *T*_2_, representing the relaxation time constant of the longitudinal and transverse components of the magnetization, respectively. Relaxation time constants vary with the tissue environment, with shorter relaxation times observed in more restricted environments. For an anisotropic tissue such as cartilage, *T*_2_ also varies with the angle of collagen alignment, relative to the static magnetic field [15] due to the intramolecular dipolar interactions of water and cartilage [3]. The inverse of *T*_2_ represents a measure of relaxation rate (*R*_2_) of the nucleus and is used as the measure of relaxation in this study. The R_2_ of each voxel is plotted as a parametric map of tissue relaxation rate, showing highest intensity for the regions with greatest relaxation rates. This information can be used to investigate the molecular environment of cartilage tissue in various states of compression and disease.

Attempts to assess load-dependent changes in cartilage water *T*_2_ *in vivo* have demonstrated a potential role for MRI [16], however the variability and resolution of *in vivo* measurements indicate a need for more precise *in vitro* measurements to enable modeling of cartilage behavior and to identify factors associated with early loss of cartilage viability.

Highly aligned and structured, cartilage is an example of a poroelastic material having a supporting collagen matrix associated with a viscous liquid component [17] that osmotically interacts with proteoglycans. As such it can be investigated and modeled using traditional poroelastic models, as used in engineering [18].

One mechanical testing technique commonly used to assess cartilage function is consolidometry. This involves application of a constant load and measuring the time dependent change in thickness or stress creep, together with the change in pressure as the sample responds. This response is determined by the osmotically active components of the cartilage as they oppose the pressure-induced egress of water from the matrix over time and is defined as the hydrostatic excess pore pressure (HEPP).

While methods are available to assess late stages of cartilage damage *in vivo* [19–22], *in vitro* mechanical testing is the only non-destructive experimental method able to assess the load bearing function in healthy and diseased cartilage. To gain maximum information about cartilage response to load and the influences of disease and stress on structural change, it is desirable to combine mechanical and image-based measurements. Before simultaneously performing MR imaging in conjunction with mechanical testing, as a means to study the complex behavior of cartilage, there is a requirement to incorporate physical measurements to enable quantification of performance under controlled mechanical load in real time. This is difficult due to the confined space of the MRI magnet and the impact on transducers of the strong magnetic field-strengths associated with the MRI technique. A number of groups have used different characteristics of the magnetic resonance signal to examine cartilage behaviour (reviewed in [23,24]). The stress in joints has been estimated using MRI imaging by others, who have reported creep-type strain and stress in cartilage [25] and joints [26,27], however neither the influence of hydrostatic excess pore pressure as a measure of the osmotic contribution to the load bearing behavior, nor the long-time dynamics of cartilage consolidation, have been well studied by MRI.

Here we describe a method that allows the MR imaging of cartilage before and after loading without the inaccuracies associated with repositioning the sample, while at the same time allowing the recording of changes in hydrostatic excess pore pressure associated with constant load. We have used a fiber-optic transducer to avoid the influence of the strong magnetic field on transducer performance within the magnetic field of the MRI. Here we describe an MRI-compatible cartilage consolidometer capable of recording time-resolved hydrostatic pore pressure and strain curves inside the confined space of an MRI magnet for the study of cartilage function.

## 2. Experimental Section

### 2.1. NMR Measurements

Measurement of cartilage consolidation in an MRI spectrometer places limitations on the construction materials, dimensions of the equipment and access to the tissue being measured. The instrument used for this work utilizes a 7 tesla (T) super-conducting vertical-bore magnet (Bruker WB300, Rheinstetten, Germany) coupled with a DRX NMR spectrometer (Bruker) and triple-axis imaging gradients (Bruker). The working region of the MRI magnet comprises a transmit/receiver coil of 20 mm diameter, which is centered within the imaging gradients (1.5 T/m) of the 1.5 m long and 50 mm working-diameter superconducting magnet bore. The sample is lowered into the MRI coil from above.

**Figure 2.**
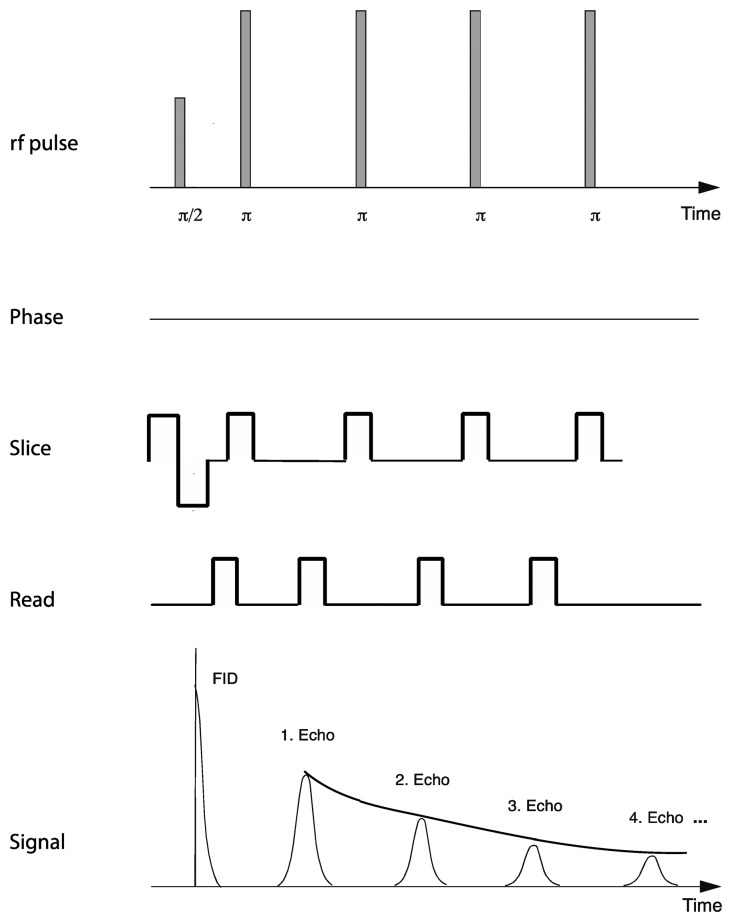
An MSME imaging sequence (simplified; for further detail see [28]). Read, phase and slice refer to the transient gradients that are superimposed on the main magnetic field to achieve spatial resolution. The relaxation time *T*_2_ is determined from the signal intensity at the echo-times following each refocusing pulse (π).

Relaxation measurements utilized a standard multi-spin-multi-echo (MSME) pulse sequence [28] in which a 90° excitation radiofrequency pulse is followed by a train of 180° refocusing pulses, separated by a constant interval or echo-time. Recording the refocused signal intensity after each echo time enables determination of the decay in signal intensity over time (Figure 2). A repetition time of 2 s was used with an echo time of 7.375 ms, which enabled acquisition of 50 equidistantly spaced echo times for each relaxation curve before the signal had decayed to the noise level of the instrument. Other acquisition parameters included 10 averages, with a repetition time of 2 s, an effective spectral bandwidth of 90 kHz, field of view 22.1 mm × 22.1 mm, a slice thickness of 1 mm and an in-plane matrix size of 192 (phase) × 256 (read), zero-filled to 256 × 256 yielding an in-plane resolution of 86.3 × 86.3 μm and a total acquisition time of 64 min.

Each voxel in an image was fitted to a single exponential using Matlab (Mathworks) to provide a map of the relaxation time throughout the sample. The inverse of the *T*_2_ relaxation time, *R*_2_, provides a relaxation map in which the faster relaxing components have higher intensity.

Each *R*_2_ map was obtained from the series of 50 *T*_2_-weighted images sampling the *T*_2_ decay curve. The *TE* dependence of the measured intensities of every individual voxel was fitted with a three-parameter exponential fit:

(1) $$S(TE)=A\cdot e^{-TE\cdot R_{2}}+B$$

where *S* is the intensity at echo time *TE*; *A* is the intensity at *TE* = 0 and *B* is a measure of average noise in the magnitude image. The three adjustable parameters of the fit are *A*, *B* and *R*_2_.

### 2.2. R_2_ Depth Profiles

In order to construct the depth profile of the *R_2_* values within a given sample, the following procedure was used. The articular surface and the bone-cartilage interface in the sample were approximated by a polynomial fit of the order of between 1 and 3, depending on the amount of curvature in the respective interface. For each voxel, the shortest distance from the voxel centre to the polynomial curve approximating the articular surface was determined [29] The thickness of the cartilage at the respective location was determined as the distance from the articular surface to the bone-cartilage interface. The relative depth (*x*) was then calculated for each voxel as the ratio of the distance to the AS to the cartilage thickness. The voxels were then grouped in histogram bins according the their *x* values. For each histogram bin, the average *R*_2_ value and the standard deviation of the *R*_2_ were calculated. This procedure was repeated for each *R*_2_ map obtained (each sample, each compression state). The resulting data were used to construct the *R*_2_ profiles.

### 2.3. Consolidometer Construction

**Figure 3.**
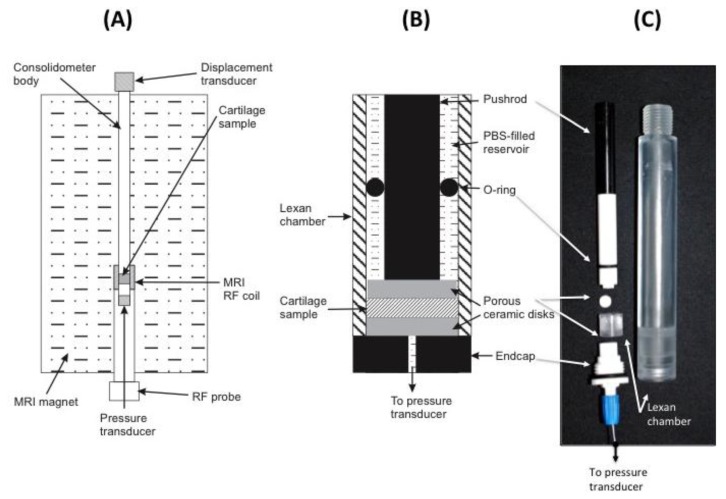
The schematic (**A**) shows the orientation of the consolidometer with respect to the superconducting MR magnet; The centre schematic (**B**) shows the arrangement of the sample chamber components; The image on the right (**C**) shows the Lexan^®^ extension and its components (from the top): the plunger; ceramic spacer; sample chamber and fiber-optic cable in the base of the sample holder. When assembled, these are screwed to the base of the aluminium consolidometer body.

To avoid MRI imaging artifacts, all material used in the construction of the consolidometer must be non-magnetic and transparent to radiofrequencies in the region of the transmit/receive coil of the instrument. The equipment constructed for this work was based around an 80 cm × 2 cm tubular aluminium housing to support all other components (Figure 3A). Attached to the top of this tube is a low voltage displacement transducer (DT; Solartron Metrology DG 2.5; RS Components, Sydney, Australia) to record the position of a regulated pneumatic 5 mm plunger for applying a known load to cartilage samples. An in-line pressure transducer (1,000 kPa, 0–100 mV; RS Comonents) the applied pneumatic pressure. A Lexan^®^ extension is screwed to the base of the aluminium housing (Figure 3B,C).

A small ceramic sample support (Macor^®^; Corning Inc., Corning, NY, USA) is screwed to the base of the Lexan^®^ extension. Figure 3C shows this sample support and the Lexan^®^ extension, which form a chamber to locate the cartilage sample and provide access from below via a small hole for a fibre-optic pressure transducer (SAMBA-201 Preclin 360 MR Special; Biopac Systems Inc., Goleta, CA, USA), incorporating a Fabry-Perot interferometer sensor [30]. Pressure calibration relied on the built-in self-calibration protocol based on atmospheric pressure. The fiber-optic cable from the sensor is held in place with an O-ring compression fitting. All components of the sample chamber are sealed by O-rings to prevent leakage of solution into the MRI magnet. Data recorded by the two transducers are transmitted via USB connection to a Windows XP personal computer and processed using Labview software (Version 7.1; National Instruments, Austin, TX, USA).

### 2.4. Tissue Preparation

Bovine patellar cartilage from 4 animals, 2–3 years old, was collected from a local abattoir and stored on ice. A battery operated drill with 10 mm diamond-tipped hole saw (DTA, Victoria, Australia), designed for ceramics and glass, was used to remove a plug of cartilage attached to underlying bone. Five samples were drilled, two from the same animal. Measurements are presented for four samples. The results from one of the duplicate samples were not of sufficiently high quality and are not presented here. To prevent air-bubble induced image artifacts, all components are immersed in a bath of physiological saline solution prior to assembly into the consolidometer. Figure 3 shows the components of the sample holder and the position of the fiber-optic transducer. Because the samples were obtained as discarded material from a commercial abattoir, the institutional ethics committee deemed that formal approval was not required for this study.

### 2.5. Software for Control of Consolidometer and Recording Results

**Figure 4.**
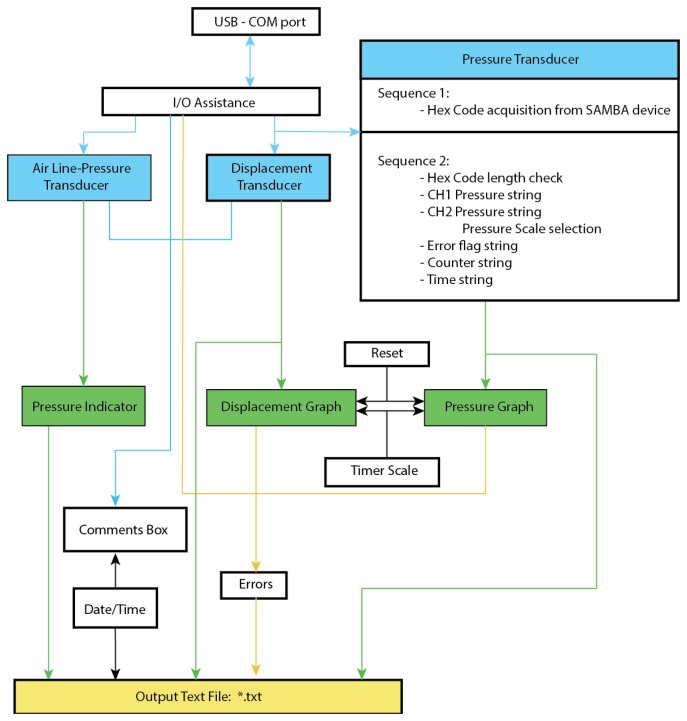
A block diagram of the components utilized in the module developed for the Labview program to interface with the signal transducers and screen output (blue and green boxes, respectively).

A Labview software routine was written to coordinate the recording of the consolidometer transducer output as shown in Figure 4. The outputs from the air line-pressure, and displacement transducers are transferred via a “personal measurement device” (PMD1208LS, RS Electronics), to the LabView program by means of a high-speed USB connection. The Labview program provides the following outputs in a graphical interface: (a) real-time graph of the transducer displacement as a function of time, showing the distance that the cartilage is compressed (in millimetres) in the time since monitoring was started (in seconds), updated every 1 s; (b) a real-time graph of the pressure as a function of time provides the hydrostatic pore pressure of the cartilage, as measured by the SAMBA transducer during the time since monitoring was started (in seconds), also updated every 1 s; (c) the real-time distance as recorded by the distance (compression) transducer in the top end of the consolidometer body; (d) a view of the pressure being applied to the system as measured by the air line-pressure transducer at the air supply point; (e) a HEX key that is transmitted from the SAMBA unit before it has been decoded, available for diagnostic work; and (f) an error display panel to warn of incorrect performance.

The following operator input options are also available for the Labview module: a comments field; an option to filter (not used in this work) the signal before graphical display, while the unfiltered signal is still recorded in the log file; a pull-down menu for selection of units used in the pressure graph display and data in the log file; a reset option that allows the clearing of data from the graphs while the system is running.

## 3. Results

### 3.1. Consolidation Curves

**Figure 5.**
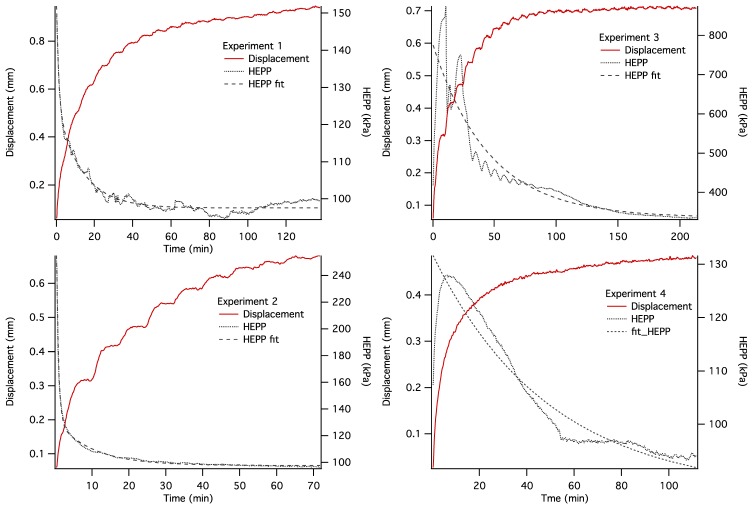
Unconstrained consolidation curves of the four articular cartilage samples showing HEPP curves (black) and the corresponding compressive displacement *vs.* time. The fluctuations of the applied pressure are an artifact of the compressed air supply shown in Figure 6. The samples were bone-cartilage plugs similar to that seen in Figure 7.

**Figure 6.**
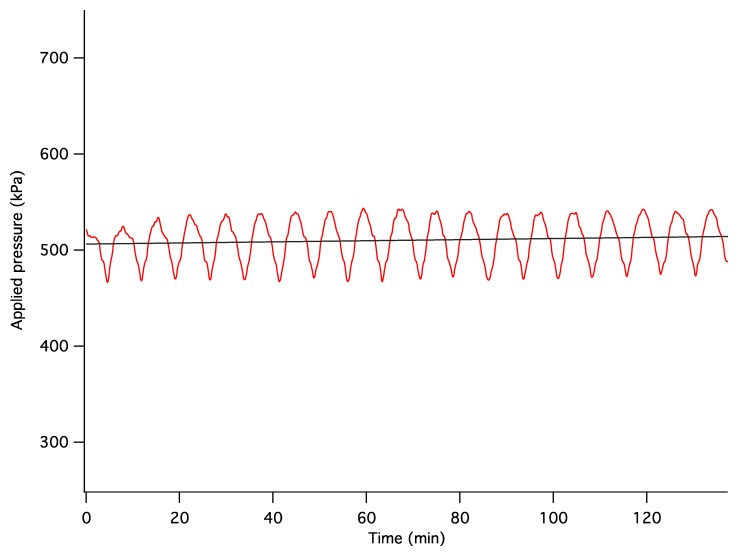
A representative plot showing the fluctuations in the supplied air pressure *vs.* time consistent with the fluctuations of the applied pressure shown in Figure 5 being an artifact of the compressed air supply.

The dynamics of unconstrained mechanical consolidation of articular cartilage samples were characterised by measuring the time dependence of two quantities: hydrostatic excess pore pressure (HEPP) and compressive displacement. These consolidation curves (hereafter referred to as the HEPP and the DT curve, respectively) were recorded for the four samples studied. Figure 5 shows the HEPP and the DT for the four articular cartilage samples. A representative example of the time dependence of the applied pressure is also shown in Figure 6; the small periodic oscillations of the applied pressure correspond to the oscillations of the efficiency of the pneumatic compressor related to pressure regulation. The compression ratio of the samples was defined as:

(2) $$C=\frac{h_{0}-\Delta h}{h_{0}}\cdot 100\%$$

where *h*_0_ and Δ*h* are the thickness of the uncompressed cartilage and the compressive displacement, respectively, measured from MRI images. Consolidometry data were not used for its calculation because the displacement-*vs.*-time curve provides information about the dynamics of Δ*h* but not about *h*_0_; therefore, it is not possible to calculate *C* on the basis of consolidometry measurements alone. The value of *C* ranged from ∼80% at the applied pressure of 50 kPa to ∼25% at the applied pressure of 500 kPa. The ratio *C* was calculated on the basis of the *average* change of cartilage thickness between uncompressed and compressed MR images. In cartilage samples with a curved articular surface or bone-cartilage interface the local compression ratio may vary across the sample due to lateral variation of the stress. In such samples the definition of *C* may be somewhat ambiguous. For this reason, *C* was used here only as an overall guide of the degree of sample compression; it was not used in data processing or interpretation. The typical excursion of HEPP over the course of consolidation typically ranged between 100 and 300 kPa, depending on the applied pressure. The approach of both the HEPP and the compressive displacement to their respective asymptotic values could be characterized as multiexponential. The HEPP curves in Figure 5 also show the line obtained using a double exponential fit. The initial buildup and subsequent decay of HEPP to 50% of the maximum value typically took several minutes, but it usually took ∼120 min for the consolidation process to be considered near complete.

### 3.2. T_2_-Weighted Images

**Figure 7.**
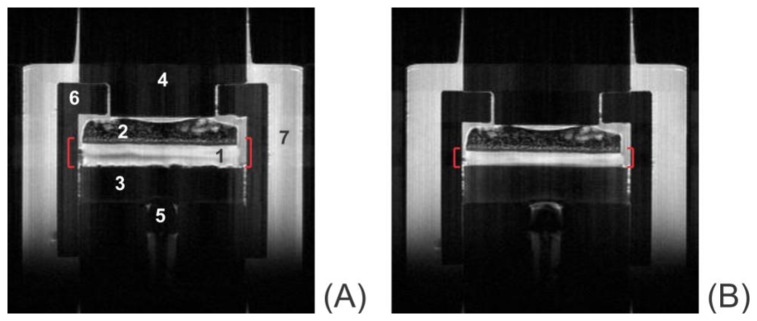
Representative *T*_2_-weighted images of bone-cartilage plugs: (**A**) uncompressed Sample 4 and (**B**) Sample 4 compressed under the applied pressure of 110 kPa. Both images were taken at the echo time of 7.375 ms. Labeled with the numerals are: (1) articular cartilage; (2) subchondral bone; (3) compression disc made of permeable ceramic; (4) compression rod; (5) Fabry-Perot pressure sensor; (6) sample housing; and (7) phosphate-buffered saline filling the compression chamber. The compressed image (**B**) was obtained in the quasistatic limit of the consolidation curve (∼2 h after the start of consolidation). The compressive displacement, C, was 64%.

Figure 7 shows typical *T*_2_-weighted images of uncompressed (Figure 7A) and compressed (Figure 7B) articular cartilage Sample 2. The images shown in this figure were acquired near the full image intensity (short echo time, *TE* = 7.375 ms). In all, 50 *T*_2_-weighted images were acquired in the same data set in order to sample the *T*_2_ decay curve: the TE values were sampled equidistantly in the range from 7.375 to 368.75 ms. The components of the consolidometer and the elements of the sample visible in the images are labeled in the figure and identified in the figure caption.

### 3.3. R_2_ Map

**Figure 8.**
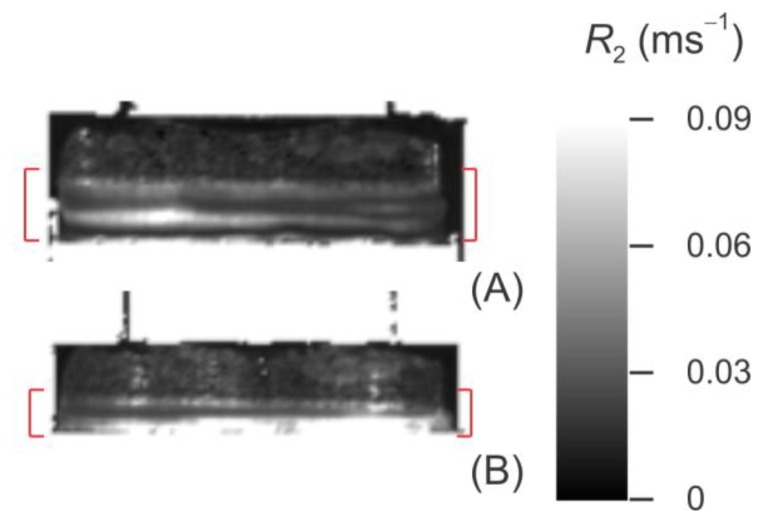
*R*_2_ maps of Sample 4. Metal and plastic components of the consolidometer, which produce no MRI signal, have been masked out. The red brackets show the cartilage before (**A**) and after (**B**) compression, with the remaining signal arising from the supporting bone. A linear greylevel scale is used, with white corresponding to *R*_2_ = 0.09 ms^−1^ and black, to *R*_2_ = 0.

Figure 8 shows the *R*_2_ maps for Sample 2, the fitted value of *R*_2_ (as defined in Equation (1)) taken as the transverse spin relaxation rate for the given voxel. For the sake of clarity, the regions that produce no MRI signal (*i.e.*, the metal and plastic components of the consolidometer) have been masked out, and only bone-cartilage plugs and PBS are shown in this figure.

### 3.4 Effect of Compression on T_2_ Depth Profiles

**Figure 9.**
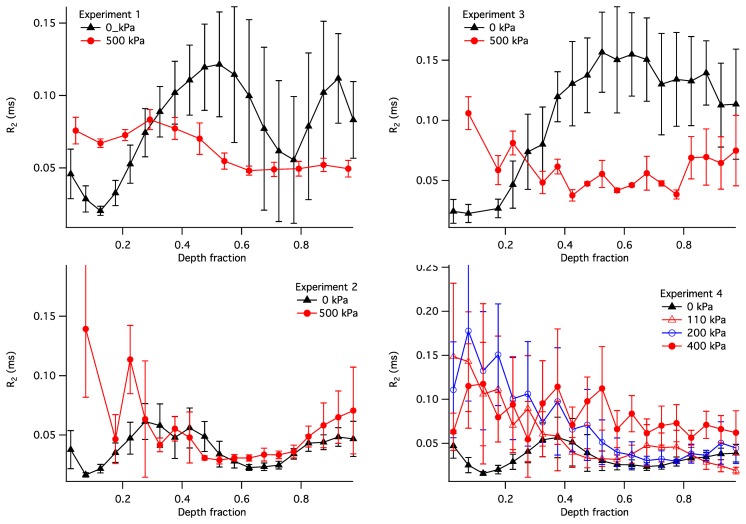
Depth profiles of the transverse spin relaxation rate (*R*_2_) in uncompressed (black plot) and compressed (red plot) articular cartilage in each of the four samples (panels correspond to the panels in Figure 5). Depth fraction is the normalised depth (relative to the cartilage surface) The “compressed” data were obtained at the load of 500 kPa for all except sample four in which a series of pressures (110, 200 and 400 kPa) were applied sequentially. In each plot, the solid line shows the average value of *R*_2_, and the error bars show the standard deviations. The plot demonstrates that static load carriage in articular cartilage involves primarily regions consistent with the superficial and transitional zones, where the greatest compression-induced change in the *R*_2_ is observed.

The observed *R*_2_ within the articular cartilage exhibited a non-uniform and compression-dependent depth profile. The uncompressed and compressed *R*_2_ depth profiles for the four samples are presented in Figure 9. In order to adequately compare the profiles in uncompressed and compressed samples, the *R*_2_ in this figure is plotted *vs.* the normalised depth (*x*): a unit-less quantity equal to the depth divided by the thickness of the articular cartilage where the cartilage surface is zero. It is used here instead of the actual depth in order to enable a comparison between compressed and uncompressed samples. Each point in the plots illustrates a distribution of the *R*_2_ values at a given normalized depth: the solid lines show the means, with error bars showing the standard deviations.

## 4. Discussion

### 4.1. Consolidation Curves

Construction of an MRI compatible consolidometer, utilising a Fabry-Perot pressure transducer that is unaffected by strong magnetic fields, enabled the recording of consolidation curves comparable to those previously reported [6,31]. While MRI micro-imaging of static cartilage has been reported previously [15], the ability to undertake biophysical measurements of cartilage under compressive load has extended the potential of the cartilage consolidation technique to contribute to our understanding of the functional behaviour of cartilage. The irregular consolidation curve observed for Sample 3 in Figure 5 can be explained by a combination of o-ring stiction and the presence of bubbles in the system.

### 4.2. Transverse Spin Relaxation Rates (R_2_) in Articular Cartilage

Transverse relaxation rate (*R*_2_) of water protons in articular cartilage is determined by two principal factors: the volume fraction of the ECM biopolymers (collagen and proteoglycans) and the degree of collagen fibre alignment. The nature of this relationship can be understood in terms of the following model.

Water in articular cartilage undergoes chemical exchange between “free” water and “bound” water (water associated with the ECM macromolecules) [3,32]:

(3) $$\text{P}+{\text{H}}_{2}\text{O}⇄\text{P}\cdots {\text{H}}_{2}\text{O}$$

where P is a biopolymer and ⋯ denotes hydrogen bonding. This exchange process is rapid on the NMR time scale; as a result, the observed spin relaxation rate of water protons is the weighted average of the contributions from the different water populations [33]:

(4) $$R_{2}=p_{F}R_{2F}+p_{C}R_{2C}+p_{PG}R_{2PG}$$

Here, *p_F_*, *p_C_* and *p_PG_* are the molar fractions of free water, water associated with collagen fibres and water associated with proteoglycans, respectively; *p_F_* + *p_C_* + *p_PG_* = 1; and *R*_2_*_F_*, *R*_2_*_C_* and *R*_2_*_PG_* are the intrinsic spin relaxation rates associated with these populations of water.

The other important factor affecting *R*_2_ is the alignment of the collagen fibres within the ECM. The intrinsic relaxation rate of water associated with collagen, *R*_2_*_C_*, follows the so-called magic-angle orientational dependence, whereby the efficiency of spin relaxation is minimised when the collagen fibre is aligned at the “magic angle” $\theta_{MA}=\arccos(1/\sqrt{3})\approx 54.7{}^{\circ}$ [3,34,35]. As a result, the weighted-average relaxation rate also follows the magic-angle dependence:

(5) $$R_{2}=R_{2}^{\text{I}}+R_{2}^{\text{A}}\left(\theta \right)=R_{2}^{\text{I}}+R_{2}^{\text{A}0}{\left(\frac{3\cos^{2}\theta -1}{2}\right)}^{2}$$

where θ is the angle between the collagen fibres and the applied static magnetic field **B**_0_ [36]. *R*_2_ on the left-hand side of Equation (5) is identical to the *R*_2_ on the left-hand side of Equation (4); both represent the experimentally measured *R*_2_ at a given physical location within a cartilage sample at a given orientation with respect to **B**_0_. *R*_2_*^I^* is the isotropic component of the relaxation rate, which is independent of the orientation of the sample relative to **B**_0_ and can be attributed to free, PG-bound and, to some extent, collagen-bound water. *R*_2_*^A^* is the anisotropic component of the relaxation rate; this component can be attributed exclusively to collagen-bound water. In other words, the *R*_2_ contributions *R*_2_*_F_* and *R*_2_*_PG_* in Equation (4) are entirely isotropic, while the contribution *R*_2_*_C_* contains an isotropic and an anisotropic term. The anisotropic component *R*_2_^A^ is non-zero only in the case of partially aligned collagen scaffold, and its amplitude is dependent both on the degree of the alignment and on the angle between the predominant direction of the alignment and the static magnetic field (*R*_2_^A0^ is the anisotropic component or relaxation rate when the cartilage is predominantly aligned with the static magnetic field).

The “free” water is the dominant water population in articular cartilage: both *p_C_* and *p_PG_* can be estimated to be ∼0.1 or less, based on studies of similar hydrated macromolecular systems [37]. However, the intrinsic spin relaxation rates of the two “bound” populations, *R*_2_*_C_* and *R*_2_*_PG_*, significantly exceed that of the free water, *R*_2_*_F_*, due to the lower molecular hydrodynamic mobility of the former. As a result, despite its relatively small molar fraction, the *R*_2_ contribution from bound water can significantly exceed that from free water. This can be seen, e.g., in references [34,35], where the amplitude of the anisotropic component of *R*_2_ within the radial zone of AC exceeds the isotropic component by a factor of 3–4. It also means that, when the cartilage sample is perpendicular to **B**_0_, the *R*_2_ value tends to vary with the depth and exhibits a zonal behaviour corresponding to the histological zones of collagen fibre alignment.

### 4.3. Interpretation of Compression-Induced R_2_ Changes

Compression of articular cartilage effects two principal changes at the microstructural level, both of which bring about a change in the *R*_2_ value. First, due to the incompressible nature of interstitial water, compression can only be achieved via the outflow of water from the sample. Therefore, compression increases the volume fraction of the biopolymers within the sample and thus serves to increase the *R*_2_. Second, sample compression can result in reorientation of collagen fibres within the ECM scaffold [12,38], which can result in changes in the anisotropic component of the spin relaxation rate. This change can be either positive or negative, depending on whether the angle between the fibres and **B**_0_ moves further away from or closer to θ*_MA_*.

In general, these two contributions to compression-induced *R*_2_ changes are superimposed and it is not possible to separate them based on the mathematical considerations alone. However, it is often possible to attribute the observed change primarily to one of these two factors, based on the knowledge of the macromolecular factors underpinning cartilage biomechanics.

As a case study, it is instructive to interpret the depth profiles of *R*_2_ shown in Figure 9. In the uncompressed samples (the black profiles), the lowest values of *R*_2_ (and therefore the lowest *R*_2_*^A^*) is consistently exhibited at the normalised depth *x* ∼ 0.1. This region of cartilage is consistent with the centre of the transitional zone of AC, where collagen is completely disordered and therefore possesses *R*_2_*^A^* = 0. With compression the *R*_2_ in these regions increased in all four samples, consistent with compression-induced loss of water in the superficial and transitional zones of the cartilage, compression-induced partial alignment of collagen fibres, or a combination of these two factors. It should be noted that some of the compression-induced evolution of the *R*_2_ profile observed in Sample 4 could be attributed to the use of the relative depth, as seen, e.g., in [39]; however, this does not appear to be a factor for the other three samples.

With the increasing depth (*x* = 0.3–0.4), the *R_2_* in uncompressed samples increases to ∼0.05 ms^−1^; this is consistent with an increased degree of collagen alignment and the consequent non-zero *R*_2_ contribution from its anisotropic component. The *R_2_* continues to increase in Sample 3 and to some extent in Sample 1, suggesting the maximum collagen alignment near the bone. These two samples are therefore consistent with the classic three-zone collagen alignment pattern (superficial, transitional and radial zones). On the other hand, in Samples 2 and 4 the *R*_2_ significantly decreases after *x* = 0.4 and reaches a secondary minimum at *x* ∼ 0.6. This suggests the presence of a secondary transitional zone near the bone.

Below the region of cartilage consistent with the primary transitional zone (*x* > 0.3), the response of the *R*_2_ profiles to compression varied markedly. Samples 1 and 3 demonstrate a decrease in *R_2_* throughout the depth range between 0.3 and 1. This can be explained as follows. Assuming that cartilage compression results in a decrease in the water content throughout the tissue, the isotropic contribution to *R*_2_ (*R*_2_*^I^*) can only be expected to rise at a given depth. However, the anisotropic contribution (*R*_2_*^A^*) is capable of decreasing if the compression results in a realignment of collagen fibres closer to the Magic Angle relative to **B**_0_. If Samples 1 and 3 possess the classical alignment pattern, then the collagen fibres in their radial zones are likely near-perpendicular to the bone in the uncompressed state. Therefore, any compression-induced realignment of the collagen fibres would increase the angle between the fibres and **B**_0_ and, according to Equation (5), would reduce the magnitude of the anisotropic *R*_2_ contribution. Such fibre realignment has also been observed experimentally from diffusion-tensor images of articular cartilage under intermediate load [14]. Therefore, fibre realignment appears to be a plausible explanation of the compression-induced decrease in *R_2_* in these two samples.

Sample 2 exhibits no significant compressive changes in the *R*_2_ profile at *x* > 0.3. A likely explanation of this observation is that the load bearing in this sample was borne exclusively by its transitional zone. An alternative explanation may be that the increase in *R*_2_^I^ due to water loss is exactly compensated by the fibre realignment-induced reduction in *R*_2_^A^; however, the scenario appears improbable.

Sample 4 shows a *R*_2_ profile in the uncompressed state that is somewhat similar to that of Sample 2. This sample was measured at three different pressures, 120, 200 and 400 kPa. All three pressures produced an increase in in *R*_2_ at *x* < 0.35, but no significant change in the *R*_2_ profiles was seen at greater depth fractons for pressures of 120 and 200 kPa. This suggests that at the lower pressures the regions corresponding to the superficial and transitional zones carried the majority of the load. At a pressure of 400 kPa, a near-uniform and significant increase in *R*_2_ is seen at *x* > 0.35. This suggests that the deeper cartilage regions in this sample contributed to the load processing at the greater loading pressures.

### 4.4. Load Processing in Articular Cartilage

As discussed above, Figure 9 reveals that the degree of compression within mechanically loaded AC is not uniform but varies between structural regions of the cartilage with different degrees of collagen alignment. The compression-induced change in the depth profile of *R*_2_ in Sample 4 suggests that, at the applied load of 100–200 kPa, the greatest compression is observed in the superficial and transitional zones of AC, while the radial zone exhibits no significant compression. This is consistent with previously published studies, where at a low compression ratio significant changes in the collagen fibre alignment were limited to the superficial and transitional zones, and significant compressive response in the radial zone was observed only at a higher compression [40,41].

The other feature evident from Figure 4 is the significant differences in the compressive response at high pressure exhibited by the deep zones (*x* > 0.35) of the four samples studied. This is consistent with the variability of collagen fibre alignment patterns observed in previous studies [12]. While the deep zone of Sample 4 exhibited a modest but significant compressive response at 400 kPa, the corresponding zone of Sample 2 exhibited no apparent response at a comparable pressure (500 kPa). This difference is interesting, especially in light of the similarity of uncompressed *R_2_* profiles of Samples 2 and 4. Furthermore, the radial zones of Samples 1 and 3 exhibited a compressive response that was significant but different to that of Sample 4. This suggests that the macromolecular scenarios involved in load processing may vary significantly between cartilage samples, even in animals of the same species and comparable age. Further investigation of these differences may be significant to the understanding of the biophysical mechanisms involved in the development of the early stages of osteoarthritis and calls for a further, in-depth study of the spatial distribution of load processing in articular cartilage.

### 4.5. MRI Consolidometry as a Tool for the Study of Cartilage Biomechanics

The limitations of this study relate to the relatively long acquisition times required for MRI measurements on a biological time-scale, the time during which an organism might experience load and for equilibration of changes to the cartilage to occur. Time resolution could be improved, at the expense of spatial resolution, using larger voxels to improve signal-to-noise ratios (SNR). Greater SNR could also be achieved with higher magnetic fields to gain better time-resolution. In this study, the maximum pressure achieved was similar to normal physiological pressures achieved *in vivo* although the results are not relevant to peak transient working loads experienced *in vivo*. The application of higher pressures would reduce the thickness of tissue available for imaging, making it difficult to reliably differentiate regions within the cartilage.

Imaging measurements could be made during the consolidation process, however, because the consolidation process is dynamic, compression of the cartilage is likely to reduce the image quality, particularly in the early stages when fluid extrusion is greatest. For our purposes, the initial and final states of the cartilage after compression were of most interest to demonstrate the ability to make measurements before and after consolidation without moving the sample within the MRI magnet.

Because the cartilage is compressed after the second measurement, it is not possible to differentiate between changes in thickness due to collagen compression from changes due to egress of water. *T*_1_ mapping in future studies may assist with this differentiation.

The successful measurement of cartilage consolidation within an MRI magnet will enable the application of other MRI techniques to further examine the dynamic structural changes accompanying load-bearing articular cartilage and disease-related changes. Examples include assessing changes in the alignment of collagen using diffusion tensor methods, imaging of bulk water flow in response to compression and the role of proteoglycan distribution, potentially measured by ^23^Na distribution. The method is also applicable to assessing treatment effects and compression cycling.

## 5. Conclusions

The construction of a MRI compatible cartilage consolidometer, for sensitive pressure measurements in the confined space and high magnetic field strength of the MRI magnet was made possible by the use of a fiber-optic pressure transducer and non-magnetic construction materials. The consolidometer enables the concurrent *in vitro* study of cartilage structure and performance while under mechanical load. The results of this study indicate that the static load is predominantly borne by the superficial and transitional zones of articular cartilage. This technique will enable characterization of cartilage tissue load-bearing performance in different states of health, as well as differences in load processing in individual animals.
